# Measurement Errors in Polymerase Chain Reaction Are a Confounding Factor for a Correct Interpretation of 5-HTTLPR Polymorphism Effects on Lifelong Premature Ejaculation: A Critical Analysis of a Previously Published Meta-Analysis of Six Studies

**DOI:** 10.1371/journal.pone.0088031

**Published:** 2014-03-03

**Authors:** Paddy K. C. Janssen, Berend Olivier, Aeilko H. Zwinderman, Marcel D. Waldinger

**Affiliations:** 1 Division of Pharmacology, Department of Pharmaceutical Sciences, Utrecht Institute for Pharmaceutical Sciences, Utrecht University, Utrecht, The Netherlands; 2 Department of Central Hospital Pharmacy, Viecuri Hospital Venlo, Venlo, The Netherlands; 3 Deptartment of Psychiatry, Yale University School of Medicine, New Haven, Connecticut, United States of America; 4 Department of Medical Statistics, Clinical Epidemiology and Biostatistics, Academic Medical Center, University of Amsterdam, Amsterdam, The Netherlands; 5 Outpatient Department of Neurosexology, HagaZiekenhuis, The Hague, The Netherlands; 6 Private Practice, Amstelveen, The Netherlands; Nathan Kline Institute and New York University School of Medicine, United States of America

## Abstract

**Objective:**

To analyze a recently published meta-analysis of six studies on 5-HTTLPR polymorphism and lifelong premature ejaculation (PE).

**Methods:**

Calculation of fraction observed and expected genotype frequencies and Hardy Weinberg equilibrium (HWE) of cases and controls. LL,SL and SS genotype frequencies of patients were subtracted from genotype frequencies of an ideal population (LL25%, SL50%, SS25%, p = 1 for HWE). Analysis of PCRs of six studies and re-analysis of the analysis and Odds ratios (ORs) reported in the recently published meta-analysis.

**Results:**

Three studies deviated from HWE in patients and one study deviated from HWE in controls. In three studies in-HWE the mean deviation of genotype frequencies from a theoretical population not-deviating from HWE was small: LL(1.7%), SL(−2.3%), SS(0.6%). In three studies not-in-HWE the mean deviation of genotype frequencies was high: LL(−3.3%), SL(−18.5%) and SS(21.8%) with very low percentage SL genotype concurrent with very high percentage SS genotype. The most serious PCR deviations were reported in the three not-in-HWE studies. The three in-HWE studies had normal OR. In contrast, the three not-in-HWE studies had a low OR.

**Conclusions:**

In three studies not-in-HWE and with very low OR, inadequate PCR analysis and/or inadequate interpretation of its gel electrophoresis resulted in very low SL and a resulting shift to very high SS genotype frequency outcome. Consequently, PCRs of these three studies are not reliable. Failure to note the inadequacy of PCR tests makes such PCRs a confounding factor in clinical interpretation of genetic studies. Currently, a meta-analysis can only be performed on three studies-in-HWE. However, based on the three studies-in-HWE with OR of about 1 there is not any indication that in men with lifelong PE the frequency of LL,SL and SS genotype deviates from the general male population and/or that the SL or SS genotype is in any way associated with lifelong PE.

## Introduction

Lifelong premature ejaculation (PE) is defined as a male sexual dysfunction characterized by ejaculation that always or nearly always occurs prior to or within about 1 minute of vaginal penetration, the inability to delay ejaculation on all or nearly all vaginal penetrations, and with negative personal consequences, such as distress, bother, frustration, and/or the avoidance of sexual intimacy [1]. In contrast, men with acquired PE have never suffered from PE but they do experience a reduction in the ejaculation time later in life, often to an estimated intravaginal ejaculation latency time (IELT) of less than about 3 minutes [2]–[4]. In 1998, Waldinger et al postulated that lifelong PE in terms of an IELT of less than 1 minute is related to genetic factors and to diminished central 5-HT neurotransmission and/or dysfunctional 5-HT_1A_ and 5-HT_2C_ receptors [5]. Although lifelong PE is not regarded as a hereditary genetic disorder, Waldinger et al [6] reported a familial occurrence of lifelong PE in first degree relatives of some male patients with lifelong PE. After the publication of the first study on the influence of 5-HTTLPR polymorphism and IELT duration in Dutch men with lifelong PE by Janssen et al [7], five rather similar studies have since been published [8]–[12].

Recently, Zhu et al. [13] published a meta-analysis on these six studies and concluded that L-alleles of 5-HTLPR polymorphism might protect men against lifelong PE risk [13]. However, since there could be gaps and differences in the way laboratory tests were conducted and there could be differences in design and methods among each of these six studies, one has to question the validity of conducting a meta-analysis with these six studies. In this context, disturbances of Hardy-Weinberg equilibrium (HWE) as indicator of a laboratory insufficiency in genetic studies on lifelong PE has already been emphasized [14]–[16].

Similarly, Yonan et al [17] have shown that lowering the magnesium concentration of the mixture of the polymerase chain reaction (PCR) resulted in a shift of the relative allele frequencies. As a result, the initial outcome (p = 0.06) of the HWE, suggestive of an association with autism spectrum disorder, had to be reconsidered and was restorated. In other words, the initially found link between 5-HTTLPR polymorphism and autism spectrum disorder, disappeared as a result of the correction of the magnesium content of the PCR. Therefore, Yonan et al, correctly concluded that higher magnesium concentrations of the PCR caused allele-dependent, non-random genotyping errors. In addition, the importance of HWE equilibrium for correct genetic research is well-known for more than a century [18]–[26].

In the current article, we show that out of the six previously published articles on 5-HTTLPR polymorphism and premature ejaculation used for the meta-analysis, laboratory data show that three studies were not in HWE and that in those three studies the deviation of HWE is due to technical insufficiencies and/or measurement errors of the PCR. As the six studies also differed in clinically relevant factors of design and methodology, it will be argued that a reliable comparison of the six studies by a meta-analysis can not be performed.

## Materials and Methods

We analyzed the six articles that were used for the meta-analysis performed by Zhu et al [13], and also analyzed the statistical calculations as described in the meta-analysis of Zhu et al [13]. For this analysis, we only used the data that were mentioned in the six articles. Based on the absolute genotype frequencies we calculated the fraction of observed and expected genotype frequencies. With these data we calculated the HWE of cases and controls. For comparison with a theoretical population not deviating from HWE (characterized by LL 25%, SL 50%, SS 25% and therefore p = 1 for HWE) we subtracted the LL, SL and SS genotype frequencies of the patients and the controls from the genotype frequencies of the theoretical population not deviating from HWE. In other words, we calculated the difference between the observed genotype percentages and the percentages of the theoretical population not deviating from HWE.

An analysis was also performed on the polymerase chain reaction (PCR) of the six studies, as far as the details of the PCR were provided by the authors. The details pertained (i) to the content of the reaction mixture, (ii) the PCR-program and (iii) the gel-electrophoresis.

Ad (i). The content of the reaction mixture included forward and reverse primers, polymerase buffer (PB), dNTPs, magnesiumchloride, concentration of the primers, polymerase concentration, amount of genomic DNA and its total volume.

Ad (ii). The PCR-program included the first step of temperature and duration of preheating followed by cycles of duration and temperature of denaturation, annealing, extension and final hold at the end of the cyclus.

Ad (iii). The gel-electrophoresis included the concentration of the gel, the applied voltage and the duration of the procedure.

For analysis of the methods and design of the six studies we noted whether the studies were performed with a stopwatch or questionnaire, whether men reported lifelong, acquired and or both PE subtypes, and whether the IELT was within or longer than 1 minute.

Statistics: Hardy–Weinberg equilibrium to check laboratory efficacy of PCR analysis was determined using a Chi-square test. The statistics were performed by three statistical programs checking eachother outcome data: SPSS 19.0 for Windows (Chicago, IL, USA), Excel from Microsoft, Review Manager from Cochrane (version 5.2). These statistical programs were used to compare allele and genotype frequencies, to reanalyse and calculate the statistics used in the previously published meta-analysis article by Zhu et al (13) , and to recalculate the Odds Ratios (ORs) in the meta-analysis article by Zhu et al (13). In addition Risk Ratios (RRs) in the meta-analysis article by Zhu et al (13) were recalculated. P≤0.05 was considered statistically significant.

## Results

### Hardy-Weinberg Equilibrium

Table 1 shows the six studies on 5-HTTLPR polymorphism and premature ejaculation. It shows the genotype frequencies (LL, SL, and SS) of both the patients and the control individuals. Three [8]–[10] of these studies showed deviation of HWE in the patients, as reflected by their p values of ≤0.05, and one study [10] also showed a deviation of HWE in the controls.

**Table 1:** 5-HTTLPR genotype frequencies in patients and controls as reported by the authors of six studies.

Author	Year of publication	Cases	Cases				Controls	Controls				
		N	P Weinberg	%	%	%	N	P Weinberg	%	%	%	P case vs. control
				LL	SL	SS			LL	SL	SS	
Janssen	2009	89	0,9707	30,3	48,3	21,4	92	0,5862	29,3	44,6	26,1	0,5657
Safarinejad	2009	82	0,0318	29,2	35,4	35,4	82	0,1116	42,7	36,6	20,7	0,0025
Luo	2011	119	0,0003	20,1	28,6	51,3	90	0,0156	27,8	34,4	37,8	0,0002
Ozbek	2009	69	0,0543	15,9	30,4	53,7	69	0,7698	17,4	53,6	29,0	0,0002
Zuccarello	2012	89	0,6217	24,7	55,1	20,2	100	0,9174	33,0	51,0	16,0	0,0787
Jern	2012	33	0,9809	25,1	39,7	35,2	33	0,9961	30,9	44,2	24,9	0,9297

Table 2 shows the frequency difference of the genotype frequencies of the six studies with the theoretical population not deviating from HWE, characterized by (the optimal) genotype frequencies of LL (25%), SL (50%) and SS (25%). For example, in the study of Safarinejad [8], SL frequency is 35.4%, which is 14.6% lower than the 50% SL frequency in the theoretical population not deviating from HWE. Similarly, in the study of Luo et al [10], the SL frequency was 28.6%, which is 21.4% lower than the 50% SL frequency in the theoretical population not deviating from HWE.

**Table 2:** Difference of the genotype frequencies of the patients and controls of six studies with an ideal genotype frequency.

Author	Year of publication	Cases	Cases				Controls	Controls			
		N	P Weinberg	25	50	25	N	P Weinberg	25	50	25
				LL	SL	SS			LL	SL	SS
Janssen (ref 7)	2009	89	0,9707	5,3	−1,7	−3,6	92	0,5862	4,3	−5,4	1,1
Safarinejad (ref 8)	2009	82	0,0318	4,2	−14,6	10,4	82	0,1116	17,7	−13,4	−4,3
Luo (ref 10)	2011	119	0,0003	−4,9	−21,4	26,3	90	0,0156	2,8	−15,6	12,8
Ozbek (ref 9)	2009	69	0,0543	−9,1	−19,6	28,7	69	0,7698	−7,6	3,6	4,0
Zuccarello (ref 11)	2012	89	0,6217	−0,3	5,1	−4,8	100	0,9174	8,0	1,0	−9,0
Jern (ref 12)	2012	33	0,9809	0,1	−10,3	10,2	33	0,9961	5,9	−5,8	−0,1

Interestingly, in the three studies which do not deviate from HWE [7], [11], [12], the mean deviation of the genotype frequencies from the theoretical population not deviating from HWE is rather low: LL (1.7%), SL (−2.3%) and SS (0.6%). In contrast, in the three studies which do deviate from HWE [8]–[10], the mean deviation of the genotype frequencies from the theoretical population not deviating from HWE is very high: LL (−3.3%), SL (−18.5%) and SS (21.8%). Importantly, in the three studies that are not-in-HWE [8]–[10], the direction of the deviation is similar, i.e., a vey low percentage of SL genotype concurrent with a very high percentage of SS genotype.

### PCR-analysis

Table 3 shows the differences of the PCR test of the six studies. It shows that the PCRs of the six studies differed from one another. Apart from the fact that five authors did not report all the relevant information of a PCR reaction mixture, it was found that there was a difference in both the forward and reversed primers, with only two studies [7], [9] using identical primers. Moreover, the six studies differed in the polymerase buffer, the concentration of the dNTPs, the magnesium chloride concentration, the absolute concentration of the primers, the concentration of polymerase, and the concentration of genomic DNA. Furthermore, the total volume of the reaction mix differed from 10 to 50 µliter.

**Table 3:** Differences of the PCR test of the six studies with regard to primers and PCR reaction mixture; FP = forward primer, RP = reversed primer, PB = polymerase buffer, dNTPs = oligonucleotides, MgCl = magnesiumchloride.

Author	Year of publication	FP 5′-3′	RP 5′-3′	PB	dNTPs	MgCl2	primers each	Polymerase	genomic DNA	Total Volume
								U	ng	ul
Janssen	2009	GGCGTTGCCGCTCTGAATC	GAGGGACTGAGCTGGACAACCAC	1 ul 10 times	0,2 mmol/L	2.0 mmol/L	0.4 uM/L	0.5	50	10
Safarinejad	2009	GGCGTTGCCGCTCTGAATGC	AGGGGACTGAGCTGGACAAC	-	10 mM	1.5 mM	2 uM	-	20	50
Luo	2011	CTGGCGTTGCCGCTCTGAAT	GAGGGACTGAGGTGGACAACCAC	-	0,25 mmol/L			1.0	100	20
Ozbek	2009	GGCGTTGCCGCTCTGAATC	GAGGGACTGAGCTGGACAACCAC	-	0,2 mmol/L	2,0 mmol/L	0,4 uM/L	1.0	100	25
Zuccarello	2012	TGAATGCCAGCACCTAACCC	TTCTGGTGCCACCTAGACGC	2.5 uL 10 times	-	-	10 uM	-	100	23
Jern	2012	ATGCCAGCACCTAACCCCTAATGT	GGACCGCAAGGTGGGCGGGA	-	-	1.5 mM	0,3 uM	1.0	50	15

Table 4 shows the specification of the polymerase used in the various studies. Five of the six studies provided the specification of the polymerase that was used in the reaction mixture.

**Table 4:** Specification of Polymerase used in Reaction Mixture.

Author	Year of Publication	Polymerase used	Firm
Janssen	2009	AccuPrime Pfx DNA polymerase	Invitrogen Life Technologies, Strathclyde, UK
Safarinejad	2009	Polymerase in: GC-Rich PCR System	Roche Molecular Biochemicals, Basel, Switzerland
Luo	2011	-	-
Ozbek	2009	Taq Polymerase	MBI Fermentas, Hanover, MD, USA
Zuccarello	2012	Taq DNA Polymerase	Roche Diagnostics, Milano, Italy
Jern	2012	Hotstar Taq Polymerase	Qiagen

Table 5 shows the PCR program. All the six studies differed in the various parameters of the PCR-program. There was a significant difference in the duration of the preheating period. In addition, two studies [8], [10] differed in the duration of the denaturation period from the four other studies. The duration of the annealing differed in two studies [7], [8] from the four other studies. The duration of extension was aberrant in one study [9]. The duration of the final hold differed significantly from 4 to 10 minutes among five studies. The number of cycles differed from 33 to 37 among five studies.

**Table 5:** Differences of the PCR test of the six studies with regard to PCR program.

Author	Year of publication	preheating	preheating	denaturation	denaturation	annealing	annealing	extension	extension	final hold	final hold	Cycles
		Min	C	min	C	min	C	min	C	min	C	
Janssen	2009	4	94,0	0,5	94,0	0	60	1	68	4	72	33
Safarinejad	2009	3	95.5	1	95,5	1	60	1	72	7	72	35
Luo	2011	5	94,0	1	94,0	0,5	61	1	72	10	72	-
Ozbek	2009	4	94,0	0,5	94,0	0,5	60	0,75	72	8	72	33
Zuccarello	2012	4	94,0	0,5	94,0	0,5	61	1	72	4	72	37
Jern	2012	15	95,0	0,5	95,0	0,5	66	1	72	-	-	35

Table 6 shows the gel-electrophoresis. It was found that only four studies [7], [9], [10], [11] provided information of the gel-electrophoresis. In these studies, the duration of the gel-electrophoresis differed significantly from 30 to 120 minutes.

**Table 6:** Differences of the PCR test of the six studies with regard to the gel electrophoresis.

Author	Year of Publication	agarose gel	agarose gel	agarose gel
		%	min	V
Janssen	2009	2,0	120	100
Safarinejad	2009	2,0	-	-
Luo	2011	-	60	100
Ozbek	2009	2,0	30	100
Zuccarello	2012	2,5	45	150
Jern	2012	2,0	-	-

Table 7 shows the differences in study design and methodology of the six studies. A stopwatch to measure the IELT was used in only two studies [7], [8], whereas the other four studies relied on questionnaire data. Most authors used an inclusion criterion of an IELT ≤60 sec in more than 90% of sexual events. However, the Ozbeck et al. study [9] also included men who ejaculated within 1 minute only in 50% of sexual events. Moreover, three studies [7], [8], [10] reported the characteristics of the investigated cohort of men, whereas two studies [9], [11] did not report on all the characteristics and one study [12] completely failed to do so.

**Table 7:** Differences of the study design and methodology of the six studies (Y = yes, N = no).

	publication	Stopwatch	IELT ≤60 sec	population
				description
Janssen	2009	Y	Y	Y
Safarinejad	2009	Y	Y	Y
Luo	2011	N	Y	Y
Ozbek	2009	N	Partially	Partially
Zuccarello	2012	N	Y	Partially
Jern	2012	N	Y	N

## Discussion

In the current study we have shown that from the six studies, used in the meta-analysis of Zhu et al [13], three studies [8]–[10] were not in HWE, as represented by a p≤0.05. By analysing the data of the six studies and comparing these data with the calculated genotype frequencies of a theoretical population not deviating from HWE, we have found that the SL and SS genotype frequencies were normally distributed in the three studies that were in-HWE [7], [11], [12]. However, they were abnormally distributed in the three remaining studies that were not in Hardy Weinberg equilibrium [8]–[10]. Most importantly, we found that the direction of this abnormal distribution was similar in all the three studies, e.g., very low SL and very high SS genotype frequencies [8]–[10]. This phenomenon has not been described previously in the genetic literature on PE. However, these findings are in line with the study of Yonan et al [17], who initially also found a low percentage of SL genotype concurrent with a high percentage of SS genotype in a study of 5-HTTLPR polymorphism and autism disorders. However, their correction of the PCR reaction mixture by increasing its magnesium concentration resulted in a change of the genotype frequency distribution.

The remarkable similarity of the deviation in the three studies (e.g., very low SL genotype frequency concurrent with very high SS genotype frequency) only becomes clear if we understand the procedure of a polymerase chain reaction (PCR), the consequences of technical insufficiencies and/or inadequate interpretation of its gel electrophoresis.

The PCR is a biochemical technique used in a biological research lab to amplify a single or a few copies of a piece of DNA towards thousands to millions of copies of a particular DNA sequence [27]. The method relies on thermal cycling, i.e., alternately heating and cooling of the reaction to induce melting of the DNA and enzymatic replication of the DNA. Primers (short DNA fragments) containing sequences complementary to the target DNA region along with a heat-stable DNA polymerase (after which the method is named) are key components in enabling of selective and repeated DNA amplification. As PCR progresses, the DNA generated is itself used as a template for replication, setting in motion a chain reaction in which the DNA template is exponentially amplified.

A basic PCR set up requires several components and reagents [28]. These components include: DNA template containing the DNA target region, two primers, Taq polymerase, deoxynucleotide triphosphates, a buffer solution providing a suitable chemical environment for optimum activity and stability of the DNA polymerase, divalent cations, magnesium or manganese ions, and monovalent cation potassium ions.

**Figure 1:**
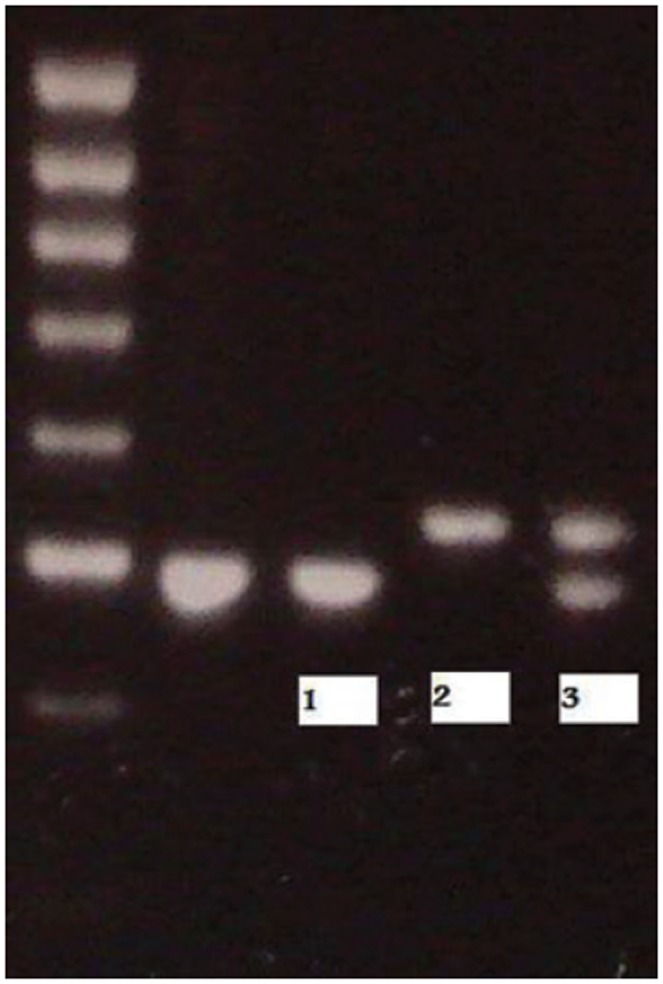
Photograph of illuminating DNA fragments on gel under ultraviolet light after electrophoresis. DNA bands in lane 1, 2 and 3 indicate successful amplification of the target sequence. The gel also shows a positive control, and a DNA ladder containing DNA fragments of defined length for sizing the bands in the experimental PCRs. Lane 1: homozygous patient for LL alleles, Lane 2: homozygous patient for SS alleles; Lane 3: heterozygous patient for LS alleles (L = long, S = short).

To check whether the PCR generated the anticipated DNA fragment (the amplimer or amplicon) “agarose gel electrophoresis” is employed for size separation of the PCR products. With this technique the amplification products are electrophoresed on 2% agarose gels at 100 Volt for 120 minutes. For this purpose the gel and running buffers need to contain the right content. In order to see the DNA fragments they need to be visualized by ethidium bromide under UV transillumination. The size(s) of PCR products is determined by comparison with a DNA ladder (a molecular weight marker), which contains DNA fragments of known size, run on the gel alongside the PCR products (see Figure 1).

Figure 1 shows a PCR product (e.g., DNA of a patient) after gel electrophoresis. For a good interpretation of this test, clear distinction of the short and the long allele is essential. However, clear distinction can be obscured by insufficiencies of the test itself. For example, a lower concentration of magnesium in the gel (or an allele specific reaction in the gel) may diminish the visibility of the long allele. As a result, the investigator will count less long alleles(L) and more short alleles (S), although these long alleles are present in the DNA content. In other words, in case of a heterozygote SL (SL in lane 3 in Figure 1) the short allele S will be visible, whereas the long allele L will be less visible. This induces the risk that the (wrong) conclusion will be made that the SL genotype frequency is low, whereas the SS genotype frequency will be high.

Our finding that in the three studies not in HWE, the SL genotype frequencies are strongly decreased concurrent with a strongly increased frequency of SS genotype, fits perfectly well with the aforementioned wrong interpretation of gel electrophoresis in case of an insufficient gel mixture of the PCR. However, it may also be the result of the work done by an inexperienced laboratory investigator with this type of lab research.

**Figure 2:**
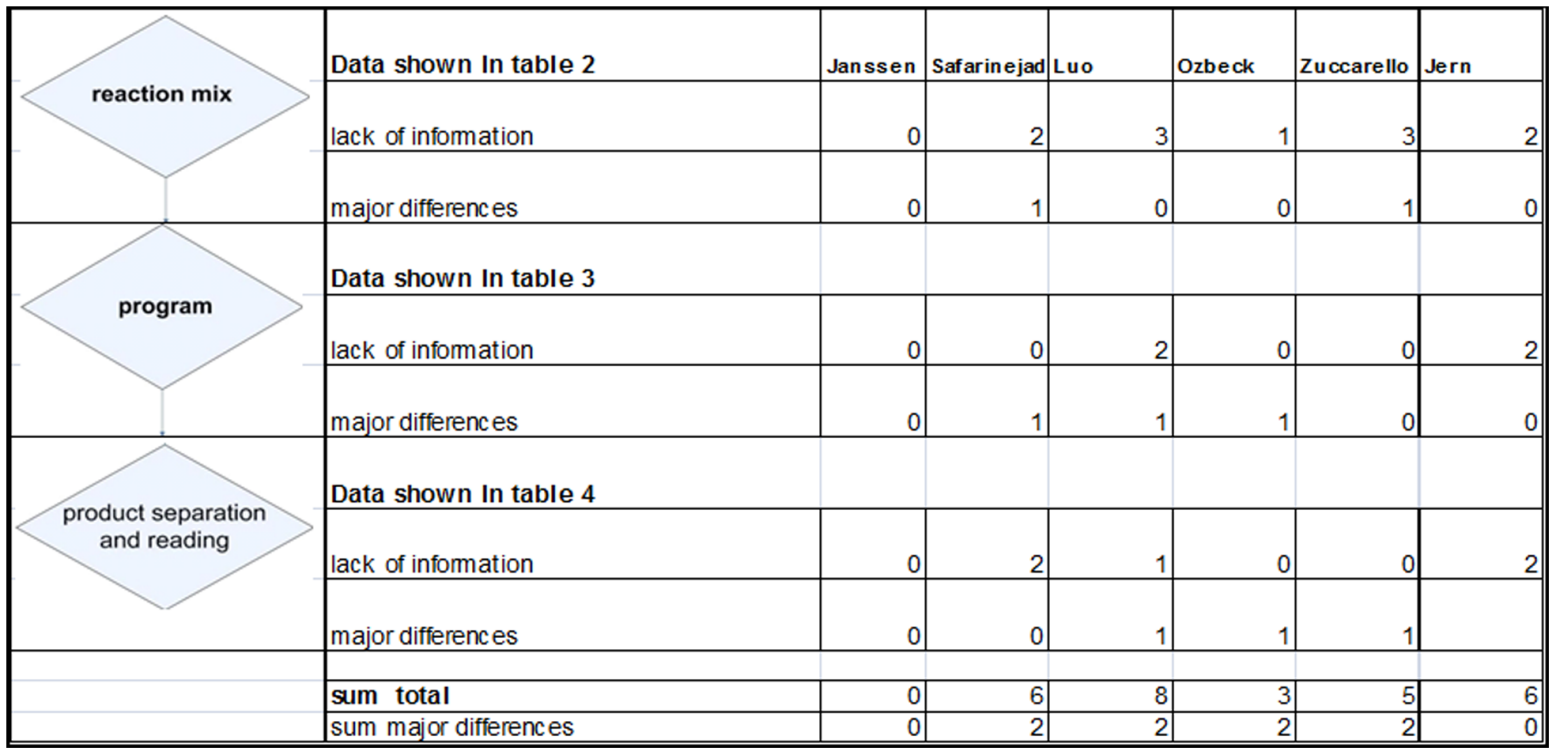
Aberrations in PCR Analysis in Six Studies. Details are represented in Tables 2–4; 0 = no aberration, 1 = one aberration; 2 = two aberrations; 3 = three aberrations.

Indeed, our additional analysis of the PCRs of the six studies, shows essential differences in the PCRs which may have influenced the outcome of these PCRs. An additional finding was that of the six articles, five authors did not provide all the required information of the PCR analysis (See Figure 2).

Notably, there appear to be important aberrations in what has been published concering in the PCR reaction mixture, the PCR program and the gel-electrophoresis. In these three phases of the PCR the most relevant aberrations are found in the studies of Safarinejad, Luo and Ozbeck [8]–[10]. For example, Luo and Ozbeck [9], [10] used a very short electrophoresis time, which may result in inadequate separation of the PCR products. Also the duration of denaturation and extension was different in the studies of Safarinejad, Luo and Ozbeck [8]–[10]. This may have resulted in inadequate extension of the DNA. Because of this gap in information, we cannot tell what the quantity of polymerase buffer was in the studies of Safarinejad, Luo and Ozbeck [8]–[10].

In summary, our analysis of genotype frequencies has shown that of the six studies those by Safarinejad, Luo and Ozbeck [8]–[10] are not-in-HWE. Additional analysis of the PCRs of the six studies shows that the major differences in the PCRs are found in the studies of Safarinejad, Luo and Ozbeck [8]–[10].

Therefore we suggest that in the three studies that were not-in-HWE and had the most significant aberrations in PCR [8]–[10], the PCR test had a preference for the short allele to become visible to the laboratory investigator. As a result, part of the heterozygotes (SL) are erratically interpreted as homozygote mutant (SS). Indeed, in the three studies not-in-HWE [8]–[10], there is a very low frequency of SL genotype and a very high frequency of SS genotype, compared to the studies of Janssen, Zuccarello and Jern [7], [11], [12] who are in-HWE, whereas the percentage of the homozygote LL genotype does not appear to be affected.

It should be noted that the study of Ozbeck et al [9] shows a marginally significant effect (p≤0.0543). Moreover, in this study the SS genotype deviation from the theoretical population not deviating from HWE is 28.7%, which is the highest in all studies.

Apart from the aforementioned technical insufficiencies of the PCR analysis and/or its interpretation, it has been found that the six studies significantly differed in clinical design and methodology. Most importantly, only two studies investigated the IELT values by using a stopwatch [7], [8]. This is rather unfortunate as prospective use of a stopwatch is a more accurate method to measure the IELT than subjective retrospective assessment of the IELT [29]–[31].

The findings of our study show that apart from a good clinical design and methodology, a correct laboratory performance and a correct interpretation of the PCR is an essential requirement for an evidence based study of 5-HTTLPR polymorphism and lifelong PE. An inadequate PCR test is a serious confounder as it may give rise to false-positive SS genotype frequencies and false-negative SL genotype frequencies. Unfortunately, this is unknown to clinicians who are not accustomed to performing a PCR themselves. Unaware of the pitfalls of an inadequate PCR test they tend to accept uncritically the (written) conclusion of the laboratory investigator. So we found that in five of the six studies essential information of the PCR has not been reported in the section materials and methods giving the impression that the authors (and also the reviewers of their manuscripts) do not consider this information important for the reader. However, we would like to emphasise that for a good understanding and interpretation of the laboratory work future genetic studies of lifelong PE and all other studies should provide all the relevant data of the PCR procedure.

As three of the six studies were not-in-HWE based on inadequate PCR analysis of DNA fragments, it may be clear that a meta-analysis cannot be performed on the six studies as they differ on the most essential procedure of genetic research. The remaining three studies that were in-HWE [7], [11], [12] and did not show dramatic PCR insufficiencies, show no significant aberations of LL, SL or SS genotype frequencies compared to the normal population. In other words, these three studies [7], [11], [12] show that the genotype frequencies of men with lifelong PE is just normally distributed. However, and interestingly, one of these three studies, showed that men with lifelong PE and with a LL genotype have a significant shorter IELT than men with SS genotype [7].

It is of note that there are indications for a geographical spread of the S-allele occurrence of 5-HTTLPR around the world. In Western Europe the S-allele frequency is about 45%, whereas in Turkey and China it is 55% and 70%, respectively [20]. According to these general data, three studies of the six articles [8]–[10] have been performed in countries with a natural higher S-allele frequency occurrence compared to Western European countries [20]. However, even when there is a natural higher S-allele frequency occurrence in non-Western European countries, this will not have any influence on our findings of the PCR test analysis.

Our view and conclusion opposes that of Zhu et al [13]. These authors who performed a meta-analysis on the same six studies, argued that a meta-analysis is allowed in spite of the fact that they are aware that some of these studies are not-in-HWE. As SS genotype may be ethnically higher in Asian populations, Zhu et al [13] separated the Asian population study of Luo et al [10] from the five other studies, which they labelled as Caucasian studies [7]–[9], [11], [12]. In addition, Zhu et al [13] calculated the pooled Odds ratio (OR) of these five single studies, as a measure of the strength of association between 5-HTTLPR gene polymorphism and lifelong PE.

Based on the L and S allele frequencies in patients and controls, Zhu et al [13] reported a low OR value for both the Asian study (OR = 0.64; CI 0.43–0.96) [10] and the five Caucasian studies [7]–[9], [11], [12] together (OR = 0.83; CI 0.80–0.98), indicating an altogether weak association of 5-HTTLPR and lifelong PE. With the also lower OR found in LL versus SS genotype frequencies in all Caucasian patients versus controls (OR = 0.88; CI 0.80–0.98), and lower OR also found in LL+LS versus SS genotype frequencies in Caucasian patients versus controls (OR = 0.83; CI 0.70–1.00), Zhu et al [13] interpreted these results as that SS genotype and/or S-allele are risk factors of lifelong PE. And therefore they concluded that LL genotype and/or L-allele might be protecting factors for lifelong PE.

**Figure 3:**
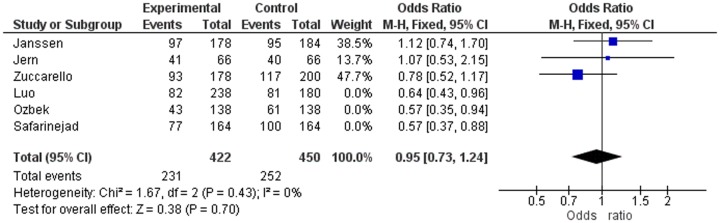
Odds Ratios of the Three Studies in-HWE.

**Figure 4:**
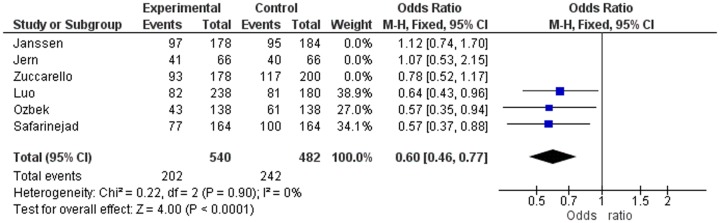
Odds Ratios of the Three Studies not-in-HWE.

In strong contrast with the study of Zhu et al [13], we have not only demonstrated but also emphasised that a very high SS genotype frequency only occurs in the three studies not-in-HWE [7], [11], [12] and that this deviation most probably is caused by misinterpretation of the gel electroforesis of the PCR analysis or a PCR reaction disturbance. Out of curiosity, we calculated the ORs of the patients and controls in the three separate studies in-HWE (Janssen, Jern and Zucarello) (Figure 3) and in the three separate studies not-in-HWE (Safarinejad, Luo and Ozbeck) (Figure 4). In addition, we calculated the pooled Odds ratios of the three studies in-HWE (Figure 3) and the three studies not-in-HWE (Figure 4) regarding allele frequency.

The ORs of the three studies in-HWE relate to Janssen, Jern and Zucarello: OR 1.12 (CI 0.74–1.70), OR 1.07 (CI 0.53–2.15) and OR 0.78 (CI 0.52–1.17), respectively. The pooled OR of these three studies in-HWE was 0.95 (CI 0.73–1.14) (Figure 3). The ORs of the three studies not-in-HWE were for Luo, Safarinejad and Ozbeck: OR 0.64 (CI 0.43–0.96), OR 0.57 (CI 0.37–0.88) and OR 0.57 (CI 0.35–0.94), respectively. The pooled OR of these three studies not-in-HWE was 0.60 (CI 0.46–0.77) (Figure 4). In other words, according to the separate ORs of the three studies in-HWE, and according to the pooled OR of these three studies together, there is no association at all between 5-HTTLPR polymorphism and lifelong PE. In contrast, as the pooled OR of the three studies not-in-HWE was 0.60 (CI 0.46–0.77) and the separate ORs of these three studies were very low, it may be erroneously concluded that there is a strong association between 5-HTTLPR polymorphism and lifelong PE.

Unfortunately, in their meta-analysis, Zhu et al [13] did not report the separate ORs of all six studies regarding the allele frequencies. Instead, as Zhu et al [13] have pooled the ORs of 5 (Caucasian) studies, including the two studies not-in-HWE (Safarinejad and Ozbek), they erroneously calculated a low OR for all five of these studies.

### Shortcomings of the Statistical Analysis of Zhu et al

Apart from our aforementioned critical analysis of the six articles, we reanalysed the data as reported by Zhu et al [13] for their OR calculations. As we were unable to replicate their outcome data, we used three statistical programs to calculate the ORs: Excel from Microsoft, Review Manager from Cochrane (version 5.2) and IBM SPSS version 19. By using the Review Manager we found a mistake made by Zhu et al [13] in their statistical calculations. Having thereby recognized their mistake, we were able to reproduce exactly their tables and figures. We found that Zhu et al [13] did calculate the OR for the study of Luo et al [10], but instead of the OR they calculated the risk ratio (RR) for the five other studies, as represented in their table 2 of the allele frequencies, in spite of the fact that they claimed to have calculated the OR of these five studies. Moreover, instead of the OR they calculated the RR for all six studies with regard to LL vs SS genotype (their figure 2) and with regard to LL+LS vs SS genotype (their figure 3). Apart from that miscalculation, we found that the legend of their figure 2 ought to refer to their figure 3, whereas the legend of their figure 3 ought to refer to their figure 2.

**Figure 5:**
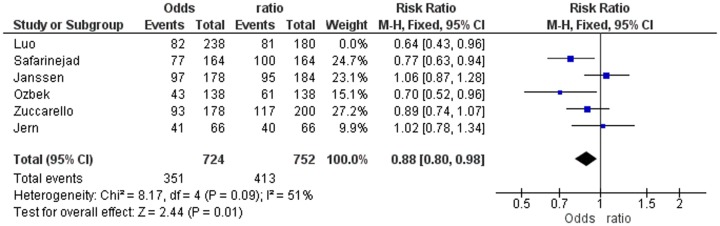
Risk Ratio of the five Studies and their pooled OR of Allelic Contrast, inadequately represented as Odds Ratio in **Table 2** in the Meta-analysis of Zhu et al [**13**].

**Figure 6:**
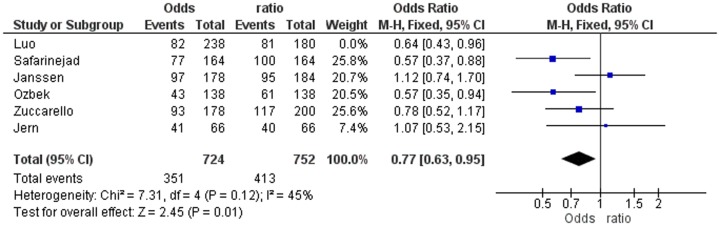
Separate ORs and pooled OR of the Six Studies regarding Allelic Contrast, as Calculated by the Current Authors using the Review Manager.

In Figure 5 we present all the data that belong to table 2 of the study of Zhu et al.[13] showing how they erroneously calculated the RR instead of the OR of the five Caucasian studies. In Figure 6 we present the separate ORs and pooled OR of all the six studies, as we have calculated them using the Review Manager.

**Figure 7:**
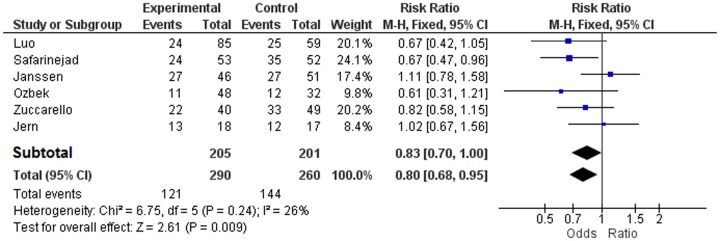
Risk Ratio LL vs SS, as misrepresented as Odds Ratio in figure 3 of the meta-analysis of Zhu et al [13].

**Figure 8:**
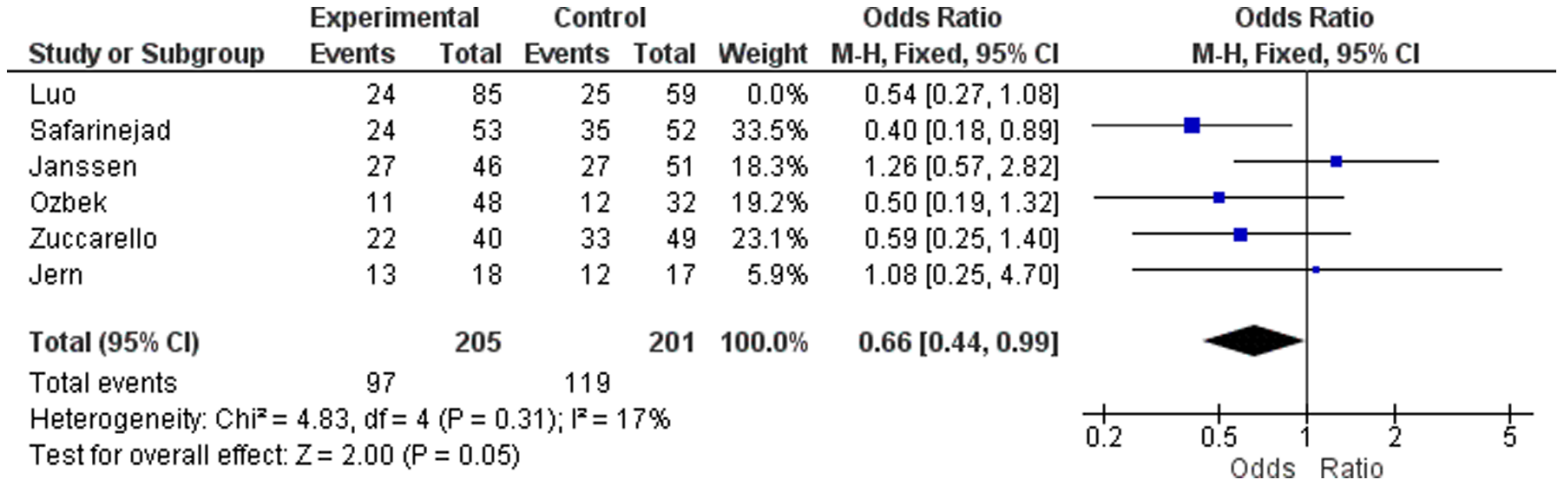
Odds Ratio LL vs SS, as calculated by us using the Review Manager.

Figure 7 shows figure 3 of Zhu et al [13], that actually represents the lifelong PE risk associated with the 5-HTTLPR gene polymorphism (LL vs SS) instead of the (LL+LS vs SS) as is erroneously represented in their article. Figure 7 shows the RR as calculated by Zhu et al [13], whereas Figure 8 shows the ORs of all the six studies with regard to the lifelong PE risk associated with the 5-HTTLPR gene polymorphism (LL vs SS). Notably, figure 2 of the study of Zhu et al. [13] contains the same miscalculations as their figure 3 (not represented here).

According to our OR calculations, the OR values of the three studies not-in-HWE are even lower than the RR values presented as ORs by Zhu et al [13]. Nevertheless, our finding of three seriously disturbed PCR tests which are at the basis of a deviated HWE and a low OR, show that these three studies are completely inadequate for use in a meta-analysis that includes three other studies with a normal PCR, - which are in-HWE -, and which have a normal OR.

## Conclusion

In our analysis of six studies that were previously used by Zhu et al [13] for a meta-analysis of 5-HTTLPR polymorphism and lifelong PE, it was found that three of these studies were not-in-HWE. In these three studies, SL genotype frequency was very low whereas the SS genotype frequency was very high compared with the three other studies that were in-HWE. As we assume that this very low SL/very high SS genotype combination is caused by an inadequate visual interpretation of the PCR test or a disturbed PCR test, we investigated the PCRs of the six studies. It was found that five of the six studies did not provide all the required information of the PCR procedure. Moreover, there were important differences in the PCR reaction mixture, the PCR program and the gel-electrophoresis, particularly in the studies that were not in HWE. Therefore, we suggest that in the three studies that were not-in-HWE, the PCR test had a preference for the short allele to become visible for the laboratory investigator. Consequently, part of the heterozygotes (SL) have erratically been interpreted as homozygote mutant (SS), leading to a false high percentage of SS genotypes. Indeed, in the three studies not-in-HWE there is a very high frequency of SS and a very low frequency of SL genotype, compared to the studies who are in-HWE, wheras the percentage of the homozygote LL genotype does not appear to be affected.

Our finding of very high SS and very low SL genotype distribution in the three studies not-in-HWE in relation to disturbances of their PCR test and/or misinterpretation of their gel electrophoresis, supports our view that understanding of the PCR procedure is pivotal for clinicians in general, and obviously for those who are involved in genetic research of 5-HTTLPR polymorphism and ejaculation. Moreover, as the outcome of a genetic research study in men with lifelong PE is heavily dependent on an adequate PCR procedure, we argue that an inadequate PCR test may behave as a confounding factor in genetic studies, particularly when the details of the PCR test are unknown to the clinician.

Notably, as the PCRs of the three studies not-in-HWE produced false SL and SS genotype frequencies, their inclusion together with the three studies in-HWE for a meta-analysis is inadequate. Our calculation of the ORs regarding allele frequencies (S and L) of patients and controls, yielded normal ORs in the three studies in-HWE and a low OR in the three studies not-in-HWE. In other words, the normal ORs of the three studies in-HWE demonstrate that there is no association at all between 5-HTTLPR polymorphism and lifelong PE.

In conclusion, our analysis demonstrate that three of the six studies who are not-in- HWE have a disturbed PCR test and a low OR, and therefore are inadequate for comparison in a meta-analysis with three other studies who are in-HWE and having a normal PCR test and a normal OR. From our analysis we also conclude that a PCR test may form a confounding factor to clinicians who do not understand the details of a PCR test, and that there is not any indication that 5-HTTLPR is associated with lifelong PE. In other words, apart from the inadequate way we have found Zhu et al [13] to have presented their data, - e.g., presenting RR data instead of the required OR data -, we conclude according to our analysis of the six studies on 5-HTTLPR polymorphisms and PE that there is no indication at all that 5-HTTLPR is associated with lifelong PE or that L alleles might protect against lifelong PE as Zhu et al [13] have erroneously concluded in their meta-analysis.
